# Impacted Mandibular Fracture: A Report of a Rare Case

**DOI:** 10.7759/cureus.38999

**Published:** 2023-05-14

**Authors:** Alladi Sneha, Sneha Pendem, Murugesan Krishnan, Pradeep Dhasarathan, Vedha Aravindan

**Affiliations:** 1 Oral and Maxillofacial Surgery, Saveetha Dental College and Hospitals, Chennai, IND; 2 Oral and Maxillofacial Surgery, Saveetha Institute of Medical and Technical Sciences, Chennai, IND

**Keywords:** sagital fracture, trauma management in maxillofacial region, miniplates, impacted fracture, mandibular fractures

## Abstract

Representing unusual fracture patterns is extremely important to understand. A 27-year-old male patient with a known history of a road traffic accident with sustained injury reported to the Department of Oral and Maxillofacial Surgery in Saveetha Dental College with pain in the left and right lower jaw region of three days duration. The patient provided a history of frontal impact in the symphysis region after a fall from a two-wheel vehicle. Clinical examination revealed a laceration of 2 cm in the chin region with bilateral pre-auricular swelling and trismus with an anterior open bite. The computed tomography scan revealed a bilateral dicapitular condyle fracture with an oblique impacted fracture of the symphysis with a displaced inferior border and left lingual cortical displacement. Apart from this, an incomplete fracture was evidenced, extending along the inferior border to the right body of the mandible. The fracture site was exposed through the laceration. The impacted mandibular fracture segments were mobilized and fixation was done using a 2 mm five-hole plate at the lower border across the sagittally split segment after placement of maxillomandibular fixation with an arch bar at the alveolar border as a part of tension banding. The oblique lingual fracture was reduced and fixed with a 2 x 14 mm bicortical screw. The primary objective of the current case report is to elucidate an unusual fracture of the mandible and discuss the management of such impacted mandibular fractures.

## Introduction

Mandibular fractures are the most common fracture of the face [1-2], accounting for about 15.5-59% of all maxillofacial fractures [3]. Among mandibular fractures, the most commonly encountered fracture is condylar fracture [4]. Being the most prominent facial bone, it often receives the maximum impact of trauma leading to direct and indirect fractures [5]. The areas of stress distribution and dissipation depend on the skeletal trabecular architecture. Often frontal injuries or injuries to the symphysis region are associated with maximum stress being transmitted to the neck of the condyle or the mandibular angle, resulting in fracture of the angle of the mandible bilaterally or of the condylar neck bilaterally. The focus of the stress concentration is also influenced by anatomical factors such as the presence of long roots of teeth and impacted teeth. When these stress levels are beyond the yield of the strength/elastic modulus of the bone, an indirect fracture occurs.

Fracture patterns in the mandible are typically well-defined and tend to follow established patterns that are determined by the force of the impact. Often, impacted fractures are commonly seen in the maxilla. Occasionally, the mechanism of injury could be different, leading to unusual fracture patterns which need precise planning and fixation of the fracture. The current case report elaborates on the pattern and management of sagittal and lingually impacted mandibular fractures.

## Case presentation

A 27-year-old male patient with a known history of seizures under treatment reported to the Department of Oral and Maxillofacial Surgery with pain in the left and right lower jaw region for three days duration. The patient had a history of a road traffic accident where he sustained and frontal impact in the symphysis region after falling from the two-wheeler. No signs of head injury were noted and the patient was conscious and oriented to time and space. The patient was transferred by the emergency medical services for primary care which involved suturing the laceration on the symphysis region and the patient was referred for further management to our centre.

On examination, the patient was conscious and oriented with a Glasgow coma scale score of 15/15. Clinical examination revealed a sutured laceration of 2 cm in the chin region with pre-auricular swelling bilaterally, trismus with anterior open bite. Palpable step-off deformity was present on the left parasymphysis region. A working diagnosis of bilateral condyle fracture with symphysis fracture was arrived at and the patient was subjected to computed tomography.

**Figure 1:**
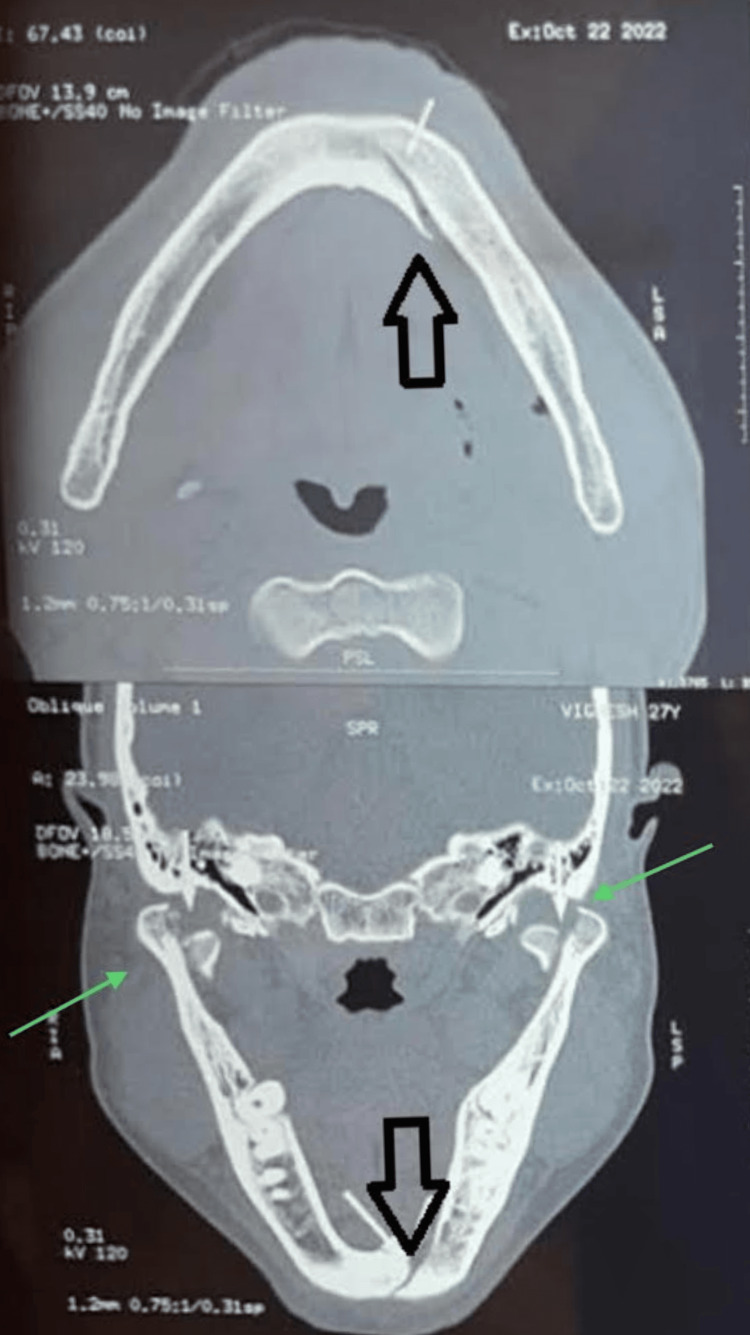
Pre-operative radiograph This image shows a pre-operative radiograph showing symphysis and a bilateral dicapitular condyle fracture.

The computed tomograph revealed a bilateral dicapitular condyle fracture with an oblique impacted fracture of the symphysis with a displaced inferior border and left lingual cortical displacement. Apart from this, an incomplete fracture was noted, extending along the inferior border to the right body of the mandible (Figure 1). Since bilaterally, the condylar fracture was within the capsule, closed reduction of the fracture was planned with maxillomandibular fixation.

The patient was planned for open reduction and internal fixation of the symphysis and closed reduction of bilateral dicapitular condylar fracture under general anaesthesia. The fracture site was exposed through the existing laceration in the chin. On exposure, the mandibular symphysis was found to be sagitally split in the lower border along with the lingual cortical plate which was extending from the right lower molar to the left lower canine. Additionally, an incomplete fracture of the buccal cortical plate was found between the central incisors.

**Figure 2:**
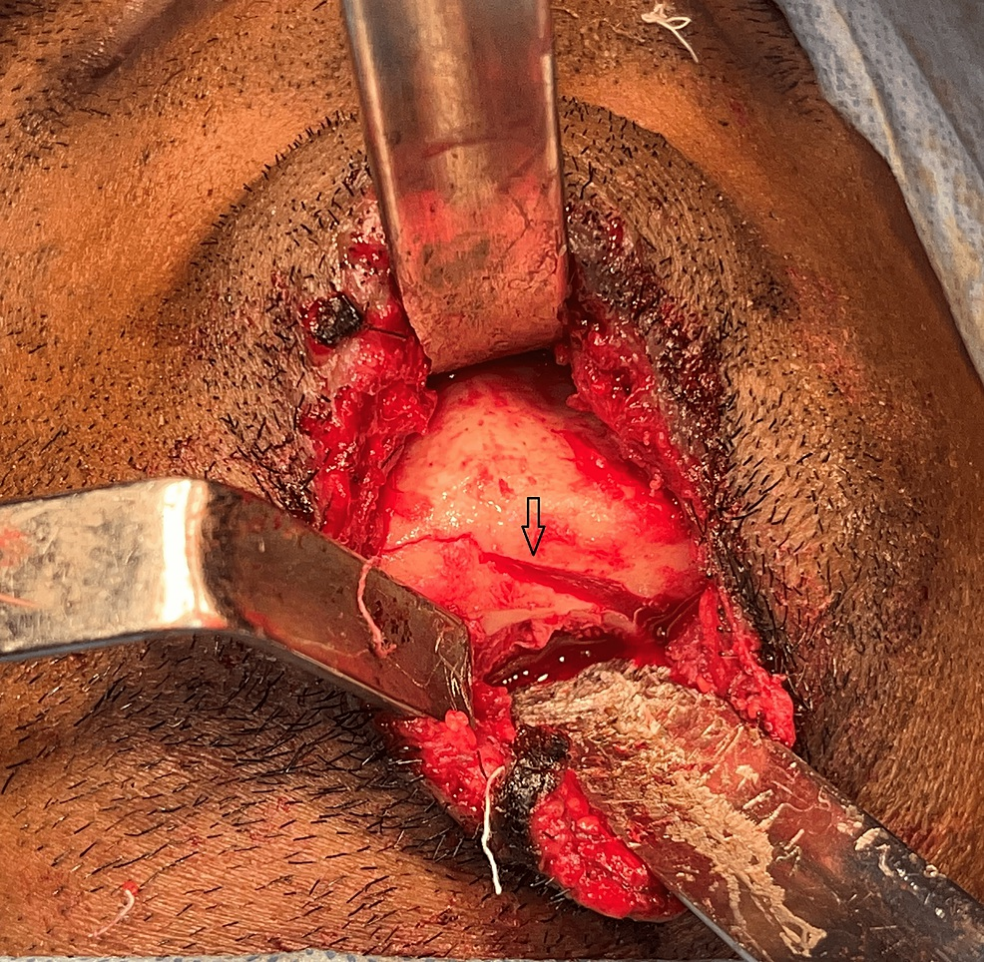
Oblique impacted fracture of the symphysis This image shows an oblique impacted fracture of the symphysis extending from the lower left canine to the right lower molar.

**Figure 3:**
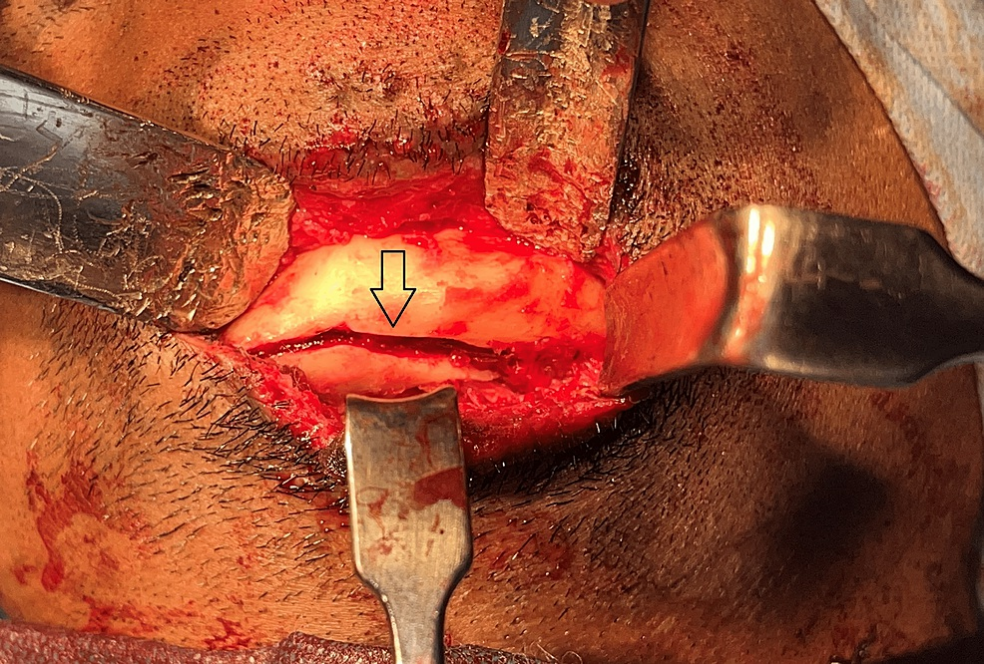
Sagittal split of the lower border of the mandible The image depicts the dis-impacted fracture showing a sagittal split in the lower border and lingual cortical plate fracture extending to the right lower molar.

**Figure 4:**
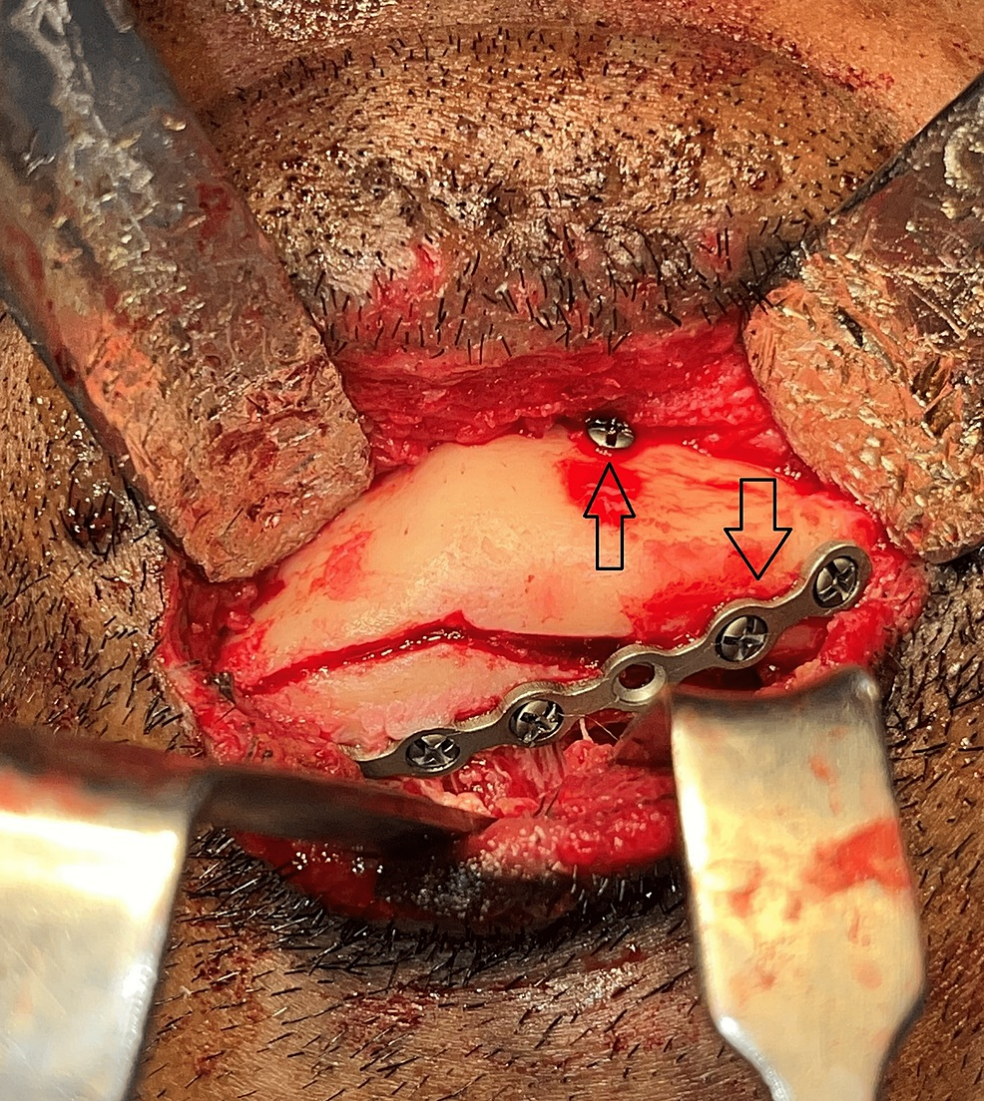
Fixation of Tte Fracture segments The image shows fixation using a 2mm five-hole plate secured using 2x8mm screws at the lower border across the sagittally split segment and with a 2 x 14 mm bicortical screw.

**Figure 5:**
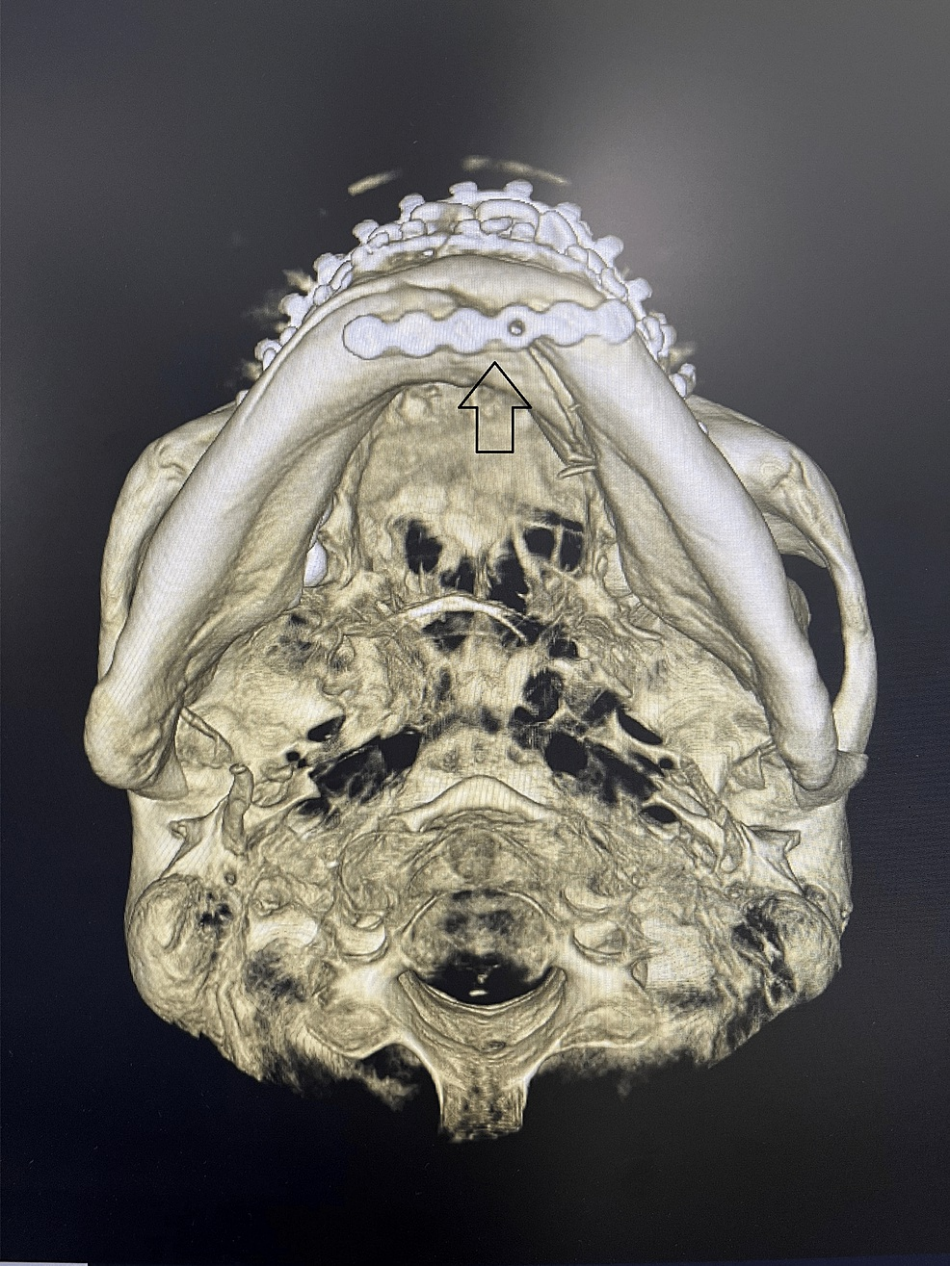
Post-operative radiograph (axial view) Post-operative radiograph depicting the reduced fracture and fixation using one 2mm five-hole plate secured using 2x8mm screws on the lower border of the mandible.

**Figure 6:**
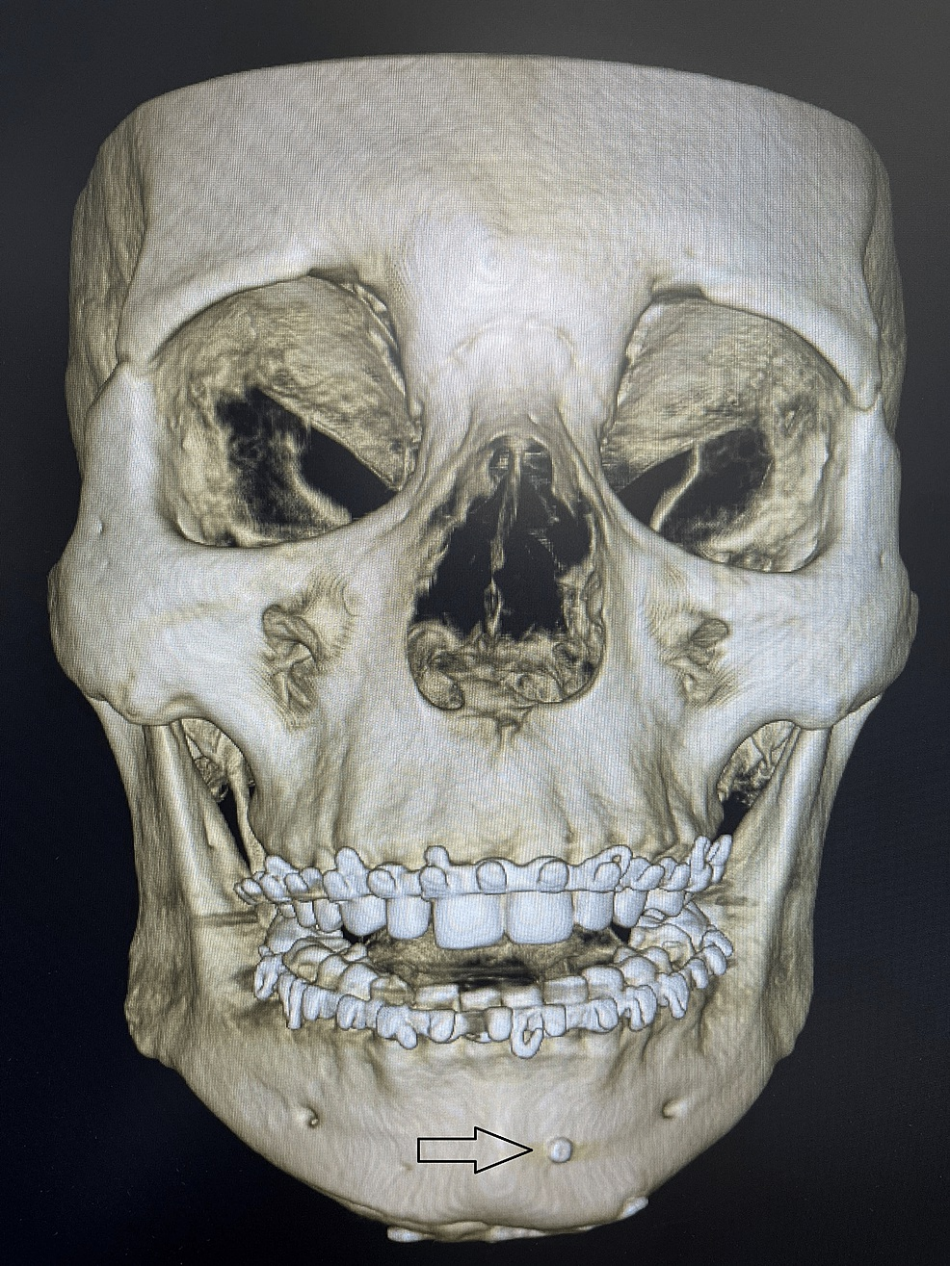
Post-operative radiograph (frontal view) This image shows the postoperative radiograph with 2 x 14mm bicortical screw fixation.

**Figure 7:**
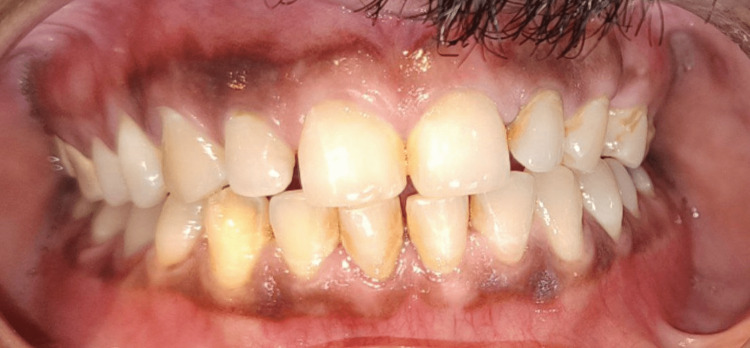
Postoperative occlusion The image illustrates the occlusion following a six-month postoperative follow-up.

The impacted mandibular fracture segments were mobilized (Figure 3) and fixation was done using a 2 mm five-hole plate and secured using 2x8mm screws at the lower border across the sagitally split segment after placement of maxillomandibular fixation with an arch bar at the alveolar border as a part of tension banding. The oblique lingual fracture was reduced and fixed with a 2 x 14 mm bicortical screw (Figure 4). The incision was closed in layers. Post-operative cone beam computed tomography was taken to evaluate the reduction to assess the anatomical orientation of the fracture segments (Figures 5-6). The patient was on maxillomandibular fixation for two weeks followed by active aggressive physiotherapy. The patient was followed up for a period of six months following the procedure, and no complications were observed during the postoperative period. The patient had pain during mouth opening in the third week which gradually reduced with active physiotherapy. After a six-month follow-up period, the postoperative occlusion remained stable (Figure 7).

## Discussion

Various factors influence the pattern of mandibular fractures which includes anatomical factors, the severity of injury, the presence of teeth and open mouth or closed mouth at the time of injury. Pertaining to forces, fractures can occur at the site of application of force or at a distant site due to indirect force called the counter-coupe fracture. The site of counter-coup injury is often determined by the weakest area of the mandible. Akama et al. [6] and Roode et al. [7] concluded that the most frequently encountered fracture in the mandible at one site is the angle of the mandible and when two or more sites are involved the site is the condyle. They also concluded that the weakest region of the dentate mandible is the condyle, the edentulous mandible being the molar region [8-9].

Considering the hunting bone concept as described by Chopra et al. [10] in guardsman fractures, the direct injury involves the symphysis followed by bicondylar fracture. The current fracture is different compared to the conventional fracture considering the bilateral symmetric diacapitular condylar head fracture with oblique symphysis fracture and an extension as greenstick fracture of the inferior border splitting the mandible sagittally until the contralateral molar. Splaying of the mandible after the bi-condylar fracture could be responsible for buccal impacted fracture with lingual obliquity. Though not many anatomical variations are found in the anterior mandible, the aetiology of oblique impacted fracture is unknown [11,12]. Yet, Pawar et al. reported two cases of comminuted mandibular fractures caused by high-speed vehicle crashes [13]

The ideal management modality of the oblique parasymphysis includes the use of rigid fixation and placement of two lag screws across to assist in bucco-lingual compression and fixation. However, in the current scenario, the oblique fracture of parasymphysis was managed by two bicortical screws and the inferior border that was split sagittally with a greenstick component until the right molar was managed by miniplate fixation. The utilization of open reduction and internal fixation for the management of fractures offers several advantages, such as decreased treatment duration, the ability to achieve anatomical reduction, and the establishment of stable three-dimensional reconstruction. Although open reduction was performed, a limitation of this case report was that the patient still required a two-week period of maxillomandibular fixation due to the condylar fracture. In a case report by Ladeinde et al, a horizontal fracture of mandibular symphysis was managed using circum-mandibular wiring producing unsatisfactory postoperative healing [14]. The closure was done in layers using polygalactin and nylon sutures [15]. Recently, antimicrobial silver nanoparticles coated suture materials have been proven to be effective in reducing the toxic effects of pathogenic organisms [16-17].

Infection due to the miniplate is a commonly reported postoperative complication [18]. However, in this particular case, no postoperative complications were observed during the six-month follow-up period. Injection of submucosal depomedrol was found to be effective in reducing post-operative pain, oedema and trismus in mandibular fractures [19]. Trypsin chymotrypsin oral combination was found to be effective in promoting healing [20]. To conclude, unusual fracture patterns of the mandible may seldom be encountered in practice. Precise pre-surgical planning with essential radiology is essential to provide the best possible treatment in a given scenario.

## Conclusions

To conclude, unusual fracture patterns of the mandible may seldom be encountered in practice. Precise pre-surgical planning with appropriate radiographs is essential for correct diagnosis and to provide the best possible treatment in a given scenario. The current case report elaborates on the aetiology, clinical findings and treatment of a rarely encountered fracture. Hence it can be concluded that miniplate fixation can be done for sagittal fracture of the symphysis with minimal complications.
